# Increases in muscle sympathetic nerve activity, heart rate, respiration, and skin blood flow during passive viewing of exercise

**DOI:** 10.3389/fnins.2013.00102

**Published:** 2013-06-11

**Authors:** Rachael Brown, Ursula Kemp, Vaughan Macefield

**Affiliations:** ^1^School of Medicine, University of Western SydneyPenrith, NSW, Australia; ^2^Neuroscience Research AustraliaSydney, NSW, Australia

**Keywords:** autonomic nervous system, muscle sympathetic nerve activity, cardiovascular, exercise, microneurography

## Abstract

The cardiovascular and respiratory effects of exercise have been widely studied, as have the autonomic effects of imagined and observed exercise. However, the effects of observed exercise in the first person have not been documented, nor have direct recordings of muscle sympathetic nerve activity (MSNA) been obtained during observed or imagined exercise. The aim of the current study was to measure blood pressure, heart rate, respiration, skin blood flow, sweat release, and MSNA (via microelectrodes inserted into the common peroneal nerve), during observation of exercise from the first person point of view. It was hypothesized that the moving stimuli would produce robust compensatory increases in the above-mentioned parameters as effectively as those generated by mental imagery and—to a lesser extent—actual exercise. Nine subjects watched a first-person running video, allowing them to view the action from the perspective of the runner rather than viewing someone else perform the exercise. On average, statistically significant increases from baseline during the running phase were seen in heart rate, respiratory rate, skin blood flow, and burst amplitude of MSNA. These results suggest that observation of exercise in the first person is a strong enough stimulus to evoke “physiologically appropriate” autonomic responses that have a purely psychogenic origin.

## Introduction

Cardiovascular responses during exercise are well-documented. Changes in autonomic activity that cause increases in sympathetic nerve activity, arterial blood pressure, heart rate, cardiac output, vascular resistance, and oxygen consumption, accompany both dynamic and isometric exercise (Delius et al., 1972; Smith et al., 1976; Lind, 1983; Victor et al., 1986; Rowell et al., 1996; Stebbins et al., 2002; Gallagher et al., 2006). There are two mechanisms responsible for these cardiovascular responses during exercise: the exercise pressor reflex, which is a reflex originating in contracting skeletal muscles, and central command, which refers to a parallel output (corollary discharge) from cortical areas involved in generating the motor command to cardiovascular control areas in the brain (Goodwin et al., 1972; Eldridge et al., 1985; Gandevia et al., 1993; Rowell et al., 1996; Sander et al., 2010).

Imagination and observation of exercise has also been shown to cause changes in the cardiovascular system, with significant changes in blood pressure, heart rate, and respiration that occur in the absence of muscle contraction or movement (Wang and Morgan, 1992; Paccalin and Jeannerod, 2000; Williamson et al., 2002; Fusi et al., 2005). These psychogenic changes have been reported during both internal and external imagery of exercise as well as observation of exercise, with significant elevations in arterial blood pressure being produced that are identical to those observed during actual exercise (Wang and Morgan, 1992; Paccalin and Jeannerod, 2000). In addition, attempted muscle contraction during complete, experimentally-induced, paralysis can cause increases in arterial blood pressure and heart rate that are comparable to those obtained during actual contractions (Gandevia et al., 1993). It is believed that autonomic activation during imagined and viewed exercise is due in part to the central processes used in motor control and is an anticipatory response to exercise (Decety et al., 1993; Fusi et al., 2005).

Given these cardiovascular changes during imagined and observed exercise, the aim of the current study is to use a first-person running video to define the autonomic responses during observed exercise. Any changes observed will be psychogenic, as they will be produced in the complete absence of changes in sensory input from the muscles. Previous studies looking at the effects of observed exercise have utilized the third person point of view only, where one is viewing someone else exercise. A first person video however, allows the subject to view the action from the perspective of the exerciser and see only what the exerciser would see, and gives the subject the feeling that they themselves are undertaking the exercise. This has not previously been reported. As observed exercise can be just as effective as exercise imagery in evoking autonomic increases in cardiorespiratory output (Fusi et al., 2005), it is anticipated that the moving visual stimuli during a first person running video should produce compensatory increases in blood pressure, heart rate, sweat release, and skin blood flow as effectively as those generated by mental imagery and to a lesser extent, actual exercise. However, what is not known is whether the increases in blood pressure reported for exercise imagery (Wang and Morgan, 1992; Williamson et al., 2002) are due to an increase in heart rate, an increase in muscle vasoconstrictor drive, or both. Accordingly, in the current study we will make direct recordings of muscle sympathetic nerve activity (MSNA) and test the hypothesis that passive viewing of first-person exercise will cause an increase in MSNA, as well as physiologically appropriate increases in heart rate, blood pressure, sweat release, and skin blood flow.

## Methods

### General procedures

Studies were performed on nine healthy subjects (6 Females, 3 males). The studies were conducted under the approval of the Human Research Ethics Committee of the University of Western Sydney, and satisfied the Declaration of Helsinki. Each subject gave informed written consent before participating in the study, and was told that they could withdraw from the experiment at any time. Subjects reclined comfortably in a chair in a semi-recumbent position with legs supported horizontally. Care was taken to ensure a calm and quiet environment with a comfortable and controlled room temperature (22°C). ECG (0.3−1.0 kHz) was recorded with Ag-AgCl surface electrodes on the chest, sampled at 2 kHz, and stored on computer with other physiological variables using a computer-based data acquisition and analysis system (PowerLab 16SP hardware and LabChart 7 software; ADInstruments, Sydney, NSW, Australia). Blood pressure was recorded continuously using finger-pulse plethysmography (Finometer Pro, Finapres Medical Systems, The Netherlands) and sampled at 400 Hz. Respiration (DC-100 Hz) was recorded using a strain-gauge transducer (Pneumotrace, UFI, Morro Bay, CA, USA) wrapped around the chest. Changes in skin blood volume, reflecting changes in skin blood flow, were monitored via a piezoelectric transducer applied to the pad of a finger; from this signal pulse amplitude was calculated using the Cyclic Measurements feature in the LabChart 7 software. A decrease in pulse amplitude was used to indicate a decrease in skin blood flow. Skin potential (0.1–10 Hz; BioAmp, ADInstruments, Sydney, NSW, Australia) was measured across the palm and dorsum of the hand, increases in skin potential reflecting sweat release.

### Microneurography

The common peroneal nerve was located at the fibular head by palpation and superficial electrical stimulation through a surface probe (3–10 mA, 0.2 ms, 1 Hz) via an isolated constant-current source (Stimulus Isolator, ADInstruments, Sydney, NSW, Australia). An insulated tungsten microelectrode (FHC, ME, USA) was inserted percutaneously into the nerve and manually advanced toward a muscle fascicle of the nerve while delivering weak electrical pulses (0.01–1 mA, 0.2 ms, 1 Hz). An uninsulated subdermal microelectrode served as the reference electrode and a surface Ag-AgCl electrode on the leg as the ground electrode. A muscle fascicle was defined as such if intraneural stimulation evoked muscle twitches of ankle or toe dorsiflexors or foot everter muscles with no radiating paresthesiae. Once a muscle fascicle had been entered, neural activity was amplified (gain 10^4^, bandpass 0.3–5.0 kHz) using a low-noise, electrically isolated, headstage (NeuroAmpEx, ADInstruments, Sydney, NSW, Australia). The position of the microelectrode tip was then adjusted manually until spontaneous bursts of muscle sympathetic nerve activity (MSNA) were identified. For identification purposes individual bursts of MSNA were generated by asking the subject to take a deep breath-hold for as long as possible. Neural activity was acquired (10 kHz sampling), RMS-processed (200 ms moving average) and analysed on computer using LabChart 7 software.

### Protocol and analysis

Recordings of resting MSNA were made over a 5-min period in which subjects breathed quietly and refrained from speaking. During this time of resting activity, subjects were looking at a static image of the initial scene. Subjects then viewed (on a large screen), a pre-recorded video of an individual running in the first person (the runner held a video camera in front of him so as to provide a moving scene as if the subject was undertaking the exercise; i.e. first-person). The video started out with a 3-min period of walking (low-level exercise) along a beach-side track, followed by a 16-min period of running up a hill, and up and down stairs, on a cliff-top running track (moderate-level exercise), finishing with 3 min of resting activity (recovery) looking out over the ocean. The subject was exposed to both the sounds of the runner and the surrounding activity, thereby providing a realistic representation of the exercise from the runner's perspective. Subjects were instructed to engage in the video as much as possible.

Peak amplitudes of MSNA, measured over consecutive 1 s epochs, coupled with sympathetic burst frequency (bursts/min), sympathetic burst incidence (bursts/100 heart beats), and the total burst activity, were measured over 4 separate periods: 3-min resting period (no imagery), 3-min walking, 16-min running, and 3-min of recovery at the end of the video. In addition, the first 3-min of the running phase was analysed separately in order to ascertain if the first 3-min of the running phase had a greater effect on the physiological parameters than the entire 16-min period. Visual inspection, coupled with auditory recognition of the neural signal, was used to identify individual bursts of MSNA. In addition, baseline was defined manually in the RMS-processed signal and the computer calculated the maximum amplitude above baseline. A beat-beat analysis was conducted for heart rate, blood pressure, skin blood flow, skin potential, and respiratory rate over each period and a mean value for each period in each subject was derived. A mean group value for each period could then be calculated and absolute changes derived. Absolute changes in skin blood flow were normalized to the individuals average resting value. Because the primary outcome was to test the hypothesis that passive viewing of exercise causes an *increase* in MSNA, heart rate, blood pressure, respiration, sweat release, and skin blood flow, for each parameter we performed a one-way paired *t*-test between the mean value obtained during running and at rest (Prism 5 for Mac, GraphPad Software Inc., USA). The level of statistical significance was set at *p* < 0.05.

## Results

**Figure 1:**
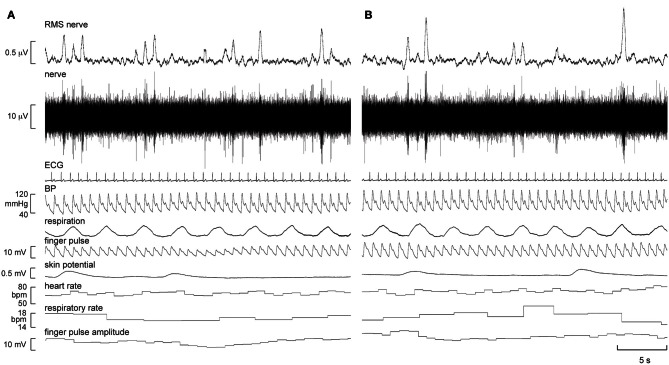
**Experimental records of muscle sympathetic nerve activity (MSNA), ECG, blood pressure, respiration, sweat release (skin potential), and skin blood flow (finger pulse) in one subject at rest **(A)** and 5 min after the start of the first-person running video **(B)**.** There was a modest increase in the number of large bursts of MSNA and a modest increase in skin blood flow during “running.”

Continuous recordings of MSNA, heart rate, blood pressure, respiration, sweat release, and skin blood flow were obtained from all nine subjects at rest, during quiet breathing, and during viewing of the first-person video of a person walking, running, and then resting. Experimental records from one subject, a 21 year-old female, are shown in Figure 1.

**Figure 2:**
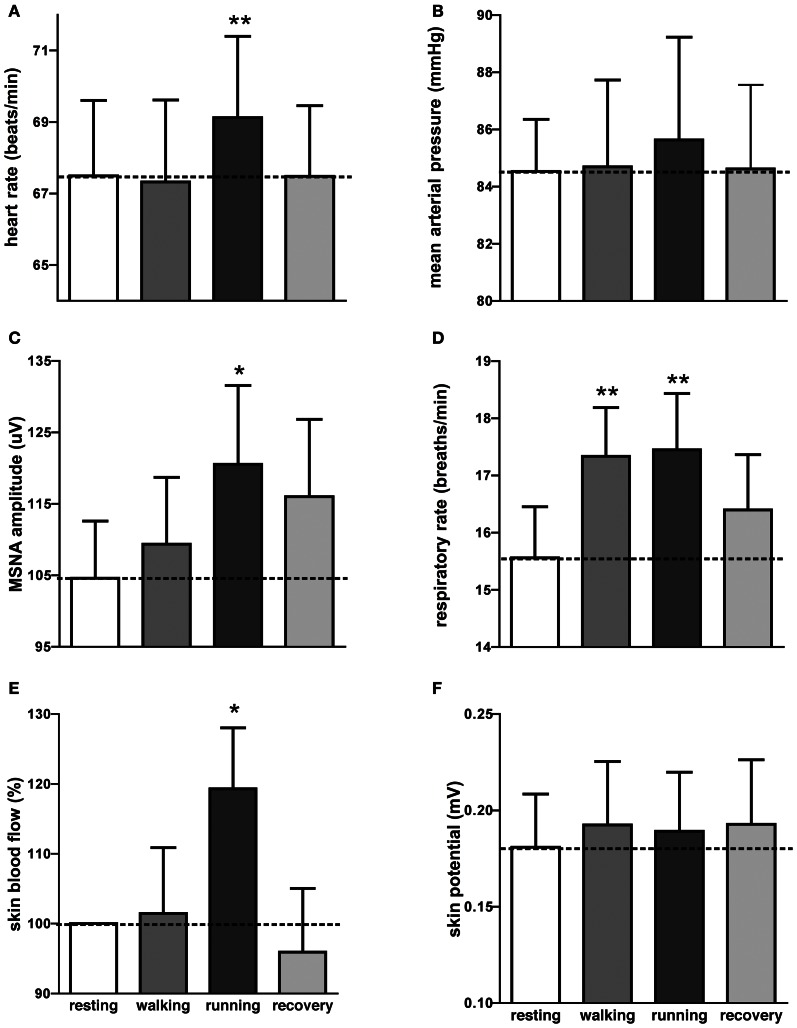
**Mean (±SE) values of heart rate (A), mean arterial pressure (B), MSNA burst amplitude (C), respiratory rate (D), skin blood flow (E), and sweat release (F) at rest, during passive viewing of first-person walking, running, and recovery.** Skin blood flow was normalized to the resting value. ^*^p < 0.05, ^**^p < 0.01.

It can be seen that there is very little difference in any of the measured parameters obtained at rest and during passive running, other than that there are more large bursts of MSNA during the running condition and a slight increase in skin blood flow. Nevertheless, on average, statistically significant increases were found between the initial resting phase and the running phase in heart rate, respiratory rate, skin blood flow, and MSNA burst amplitude. Mean values for heart rate, mean arterial pressure, MSNA burst amplitude, respiratory rate, skin blood flow, and skin potential are shown for the different phases in Figure 2. Heart rate increased by about 2 beats per minute during the running period, which is very small albeit significant. There were no significant changes in MSNA burst frequency (24 ± 3 bursts/minute at rest, 24 ± 3 bursts/minute during exercise), burst incidence (37 ± 4 bursts/100 heart beats at rest, 38 ± 4 bursts/100 heart beats during exercise), or total burst activity (18.8 ± 1.4 mV at rest, 21.7 ± 1.9 mV during exercise). In addition, there were no significant changes in mean arterial pressure, respiratory depth, or sweat release (skin potential). Finally, there were no differences to the results whether the observed running phase was averaged for the entire 16 min or just the initial 3 min.

Interestingly, an increase in respiratory rate of about 2 breaths per minute also occurred during the walking phase, meaning that even passive viewing of very low-level exercise caused a “compensatory” increase in respiration. However, when asked at the conclusion of the experiment no subject reported perceiving any level of exertion while watching the video.

## Discussion

This study has shown that observation of exercise in the first person produced statistically significant increases in cardiovascular and respiratory function. Compared to the period of rest immediately prior to the video being shown, the period of running caused significant increases in heart rate, respiratory rate, skin blood flow, and MSNA burst amplitude; an increase in respiratory rate also occurred during the period of walking that preceded the running sequence, which we believe provides evidence that the subjects were engaged in the first-person video. In addition, this is the first study to have directly recorded MSNA during passive viewing of exercise. While there were no increases in MSNA burst frequency, burst incidence, or total burst activity, there were statistically significant increases in MSNA burst amplitude from resting to observed running. This increase in burst amplitude may be responsible for the minor increase in sympathetic activity that was observed.

While it appears this is the first study to examine the effects of observation of exercise in the first person, as opposed to the third person, and while the observed increases are statistically significant, physiologically they are very small, especially the changes in heart rate and respiratory rate. Nonetheless, we did see increases, albeit small. This may be the reason therefore, that blood pressure did not increase; the increase in heart rate and respiration were not enough to drive up blood pressure. In addition, as the exercise is only observed, perhaps the lack of a contracting muscle, and therefore a lack of exercise pressor reflex is the reason for only a minor increase in heart rate and no increase in blood pressure. It is thought that central command has a greater effect on heart rate during static exercise and blood pressure increases are due to the pressor reflex response (Mitchell, 1990; Williamson et al., 1996). Therefore, it would appear that central command is the only mechanism playing a role in our increases, thereby explaining our lack of blood pressure response.

Increases in cardiovascular and cardiorespiratory output during observed exercise in the third person have been found in previous studies, with Paccalin and Jeannerod (2000) finding an increased respiratory rate in subjects observing an actor perform effortful action, while Calabrese et al. (2004) found increases in heart rate and respiratory rate during viewing of a rowing race. While the increases in these two studies are also small, they are greater than in our study, indicating that viewing exercise in the third person is a stronger stimulus on the autonomic nervous system than is viewing exercise in the first person. While subjects were asked to engage in the video, perhaps a lack of seeing the exercise being performed means the subject is not as engaged physiologically. Interestingly, Calabrese et al. (2004) also studied the same subjects with imagination of a rowing race, and found the increases in heart rate and respiration to be far greater than those found during viewing of the actual exercise.

Autonomic changes during imagined exercise, in both the first and third person, have also been found in previous studies, but none of these incorporated direct measures of sympathetic outflow to muscle. Wang and Morgan (1992) compared internal (first person) vs. external (third person) imagined exercise and found increases in blood pressure comparable to actual exercise for both forms of imagery. Interestingly, it was found that responses to internal imagery were stronger than those to external imagery. Fusi et al. (2005) found an increased respiratory rate during first-person imagined walking at different speeds, while Decety et al. (1993) found that both heart rate and respiratory rate increased during mental simulation of leg exercises at two different loads; these increases were proportional to the amount of simulated exercise. Furthermore, Williamson et al. (2002) found increases in both heart rate and blood pressure in hypnotized subjects during imagined handgrip. However, it was only those in the high hypnotizability group who exhibited increases; there were no significant changes in the low hypnotizability group. One would have expected, therefore, that our study—using first person viewed exercise—would have yielded stronger physiological changes. However, in a previous study using emotionally-charged images to evoke physiological responses, we found that the passive viewing of these images caused no effector organ responses, presumably due to a lack of cognitive stress (Brown et al., 2012). This may hold true for the current study, in that passive viewing of first person exercise involves only minor cognitive effort or attentiveness. Nevertheless, despite this apparently low cognitive load, there were significant cardiovascular responses that are physiologically appropriate during real exercise: MSNA amplitude, heart rate, respiratory rate, and skin blood flow all increased, and these changes occur during actual exercise. Perhaps what our study points to is the significant cardiovascular responses that can occur when one is fully engaged mentally and emotionally in a task. Indeed, it has been suggested that intense emotional arousal plays a significant role in the onset of acute coronary events, such as sudden cardiac death and myocardial infarction (Mittleman et al., 1995; Gabbay et al., 1996). Even watching an important football match on television can lead to such emotional excitement that myocardial infarction can occur (Wilbert-Lampen et al., 2008). Evidently, our first-person video of a person running, despite the actual runner exerting himself during the run, was not sufficiently strong to evoke larger increases in heart rate, respiratory rate, skin blood flow, sweat release, or MSNA.

While the study compares low-level exercise (walking) and moderate-level exercise (running) with an initial resting period (subject looking at a static image of the initial scene), no comparisons were made with third-person observed exercise, imagined exercise, or actual exercise. While this may be a limitation of the study, significant, and appropriate increases from resting activity to observed exercise were seen nonetheless. This leads to the question, that if third-person observed exercise and imagined exercise cause greater cardiovascular and cardiorespiratory increases than first-person observed exercise, would MSNA increases also be greater for these passive forms of exercise? Further studies undertaking direct comparisons between different forms of passive exercise would be beneficial.

## Conclusions

We have shown, for the first time, that passive viewing of exercise causes a significant increase in MSNA, together with significant and physiologically appropriate increases in heart rate, respiratory rate, and skin blood flow. It remains to be seen whether a more engaging first-person video would evoke stronger autonomic responses.

### Conflict of interest statement

The authors declare that the research was conducted in the absence of any commercial or financial relationships that could be construed as a potential conflict of interest.
